# The importance of herbal medicine use in the German health-care system: prevalence, usage pattern, and influencing factors

**DOI:** 10.1186/s12913-019-4739-0

**Published:** 2019-12-10

**Authors:** Alexandra N. Welz, Agnes Emberger-Klein, Klaus Menrad

**Affiliations:** Technical University of Munich, TUM Campus Straubing for Biotechnology and Sustainability, Chair of Marketing and Management of Biogenic Resources, Weihenstephan-Triesdorf University of Applied Sciences, Petersgasse 18, 94315 Straubing, Germany

## Abstract

**Background:**

Prevalence rates for herbal medicine (HM) have been increasing worldwide. However, little is known about prevalence, user characteristics, usage pattern and factors influencing HM usage for the general German population.

**Methods:**

A nationwide online survey on HM usage was conducted in Germany. The 2906 participants were categorised into three groups: the ones who used HM in the last 12 months, the ones who did not use HM in the last 12 months but in their lifetime, and the ones who did not use HM until now. Data were analysed by descriptive statistics, Chi Square tests and binary hierarchical logistic regression analyses.

**Results:**

Prevalence rates of HM were found to be very high for the general German population. Self-medication appeared as a common praxis, when at the same time HM users responded that they do not inform their physician about it, rate their knowledge about HM as somewhat poor, and use the internet as the most frequent source of information. The HM user in the last 12 months was found to include people that were more likely female, highly educated, privately insured, employed, and engaged in a more health-oriented lifestyle. While certain sociodemographic- and health-related variables influence HM usage vs. non-usage, they explain variance only to a limited extent. For distinguishing the user in the last 12 months vs. the less recent user who did not use HM in the last 12 months, ratings on different reasons for HM usage were found to perform better as predictors than sociodemographic- and health-related variables.

**Conclusions:**

This study demonstrated that HM usage plays an essential role in the German health-care system. Furthermore, the HM usage pattern may be potentially harmful for patients, as it included self-medication, little knowledge on interaction- and side-effects of HM, and a lack of communication with physicians about the usage. Moreover, prediction of HM usage in the previous year is impacted by variables beyond conventional sociodemographic- and health-related ones. In view of the high prevalence rates of HM in Germany, medical as well as health service providers should be aware of these issues.

## Background

Usage of complementary and alternative medicine (CAM) has been increasing worldwide during the past decades [1]. Herbal medicine (HM) was often found to be among the most popular and strongest growing forms of CAM, but as discussed in the literature HM prevalence rates show a broad variety (6–48%) in EU countries [2]. Three main reasons may explain this broad range of prevalence rates: First, country-specific variations including different cultural habits, legal details, and health insurance policies [2–5]. Second, samples that were used in previous studies often differed markedly in terms of whether or not the general population is considered, or if the sample is confined to specific subgroups such as pharmacy visitors, pregnant women, elderly people, cancer patients or people with chronic diseases [6–10]. Third, a somewhat unclear and non-uniform definition of HM was often used in the literature, e.g., HM was summarised with other “natural health products” [11] or dietary supplements such as mega-vitamins [12, 13]. These three issues may at least in part explain the strong variations in HM prevalence rates, and as such could of course also influence results on user characteristics, use-related variables, and factors predicting HM usage. Therefore, as emphasized in a review paper by Eardley et al. [2], it would be important to examine the user and use-related behaviour per country and per CAM-treatment on the basis of a clear, well-defined terminology. While previous work reported HM specific results for several countries [11, 13–15], to the best of our knowledge data on HM user characteristics, use-related variables, and predictors of HM usage for the general population in Germany have not been reported recently. Such data would be interesting for Germany in particular, because HM prevalence for the general population rates soared from ~ 50% in 1970 to 70% in 2010 [16], and were found to be very high compared to other countries [14, 15, 17]. Also, yearly reports of the German Medicines Manufacturers’ Association showed steadily increasing turnover and sales volume figures for HM products in Germany [18, 19].

In this work, we undertook a nationwide online survey regarding HM usage in the general population in Germany. We report prevalence rates, comprehensive sociodemographic and health-related characteristics related to HM usage, and compare the various user types. Furthermore, we provide insights in users’ aims and their pattern for HM usage, in addition to an examination of influencing factors for HM usage. Given that there is little population-based data in Germany and prevalence rates for HM are extremely high, our findings support the important task of establishing a better understanding of the role that HM plays in the German health-care system as well as the variables influencing the use of it. Such knowledge is critical for medicinal professionals, health-care providers as well as health-service and health-policy decision makers.

## Method

### Study design and data collection method

HM was defined as all plant-derived products including their natural form, as well as pills derived from extracts. We considered the general population in a cross-sectional study and not a specific illness group because guided by literature results we hypothesised that HM is not only used to treat an illness, but also to promote health or for preventive measures [20, 21]. In order to provide a representative picture of the general German population (18+), quota sampling was used to ensure that the sociodemographic characteristics of the participants reflected the statistical distributions of gender, age, federal state, size of household and residence in Germany. We performed an online survey using a standardised questionnaire (see below for more details) in January and February 2018 in Germany, which was the timeframe required to fulfil the requested quotas. The survey was conducted via an online panel of a market research institute, who recruited all participants. The criteria for participating in the survey were being at least 18 years old and German speaking. Before starting the questionnaire, the participants were informed about the purpose of the study and its privacy policy, and gave their consent. Participation was voluntary and monetarily compensated via the market research institute.

The online survey was completed by 3394 individuals. We carefully screened the data to ensure a valid and reliable dataset, following the criteria for data clearing provided by Schendera [22]. The remaining sample included 2906 participants. We note that this study was approved by the Ethics Commission of the Faculty of Medicine, Technical University of Munich on 08th January 2018.

### Questionnaire design

For the purpose of this survey, we developed a questionnaire (see Additional file 1) for which we mainly used established items and scales applied in previous studies, for example out of the survey instrument used for the National Health Interview Survey (NHIS) in the US [23]. Some of the items were adapted to country-specific circumstances, for example the list of herbs available in Germany. Furthermore, we developed items based on the results of our recent qualitative study, such as the items addressing the reasons of usage and the sources of information [24]. The entire questionnaire was reviewed critically by all authors as well as two research assistants. Subsequently, an online pretest (results not included in the final dataset) was performed to review the timing, clarity and general understanding of the questionnaire. Through all these steps, the questionnaire has been updated and improved. This careful process was necessary to ensure a valid, reliable and credible measuring instrument.

### Questionnaire sections and items

The first section of the questionnaire contained HM use-related questions: At the very beginning, in line with the procedure of the NHIS in the US [23], it was asked whether HM was ever used and – if yes – whether it was used in the last 12 months with the question: “Have you ever (question 1) respectively in the last 12 months (question 2) used natural herbs for your own health or treatment?” People who answered with “*yes”* received further questions regarding the following issues: aims of usage (treat illness, prevent illness, promote health), sources of information and trust in sources, self-perceived knowledge about HM (6 items on 5 point Likert scale ranging from very poor (1) to very good (5)), reasons for HM usage (13 items on 5 point Likert scale ranging from strongly disagree (1) to strongly agree (5)), whether or not participants use HM in self-medication, and whether or not they inform their physicians about the use. Furthermore, participants had to choose out of 23 possible indications for which they use HM and rate the perceived helpfulness (not at all, somewhat and a great deal). They also had to choose herbs they had used out of a list containing 26 herbs.

Section 2 addressed health-related questions, about the self-perceived health status (very poor, poor, fair, good, very good), chronic diseases (yes/no), as well as questions about physical activity (days per week), smoking (current, occasional, former, non), alcohol consumption, and whether or not specific preventive health-activities (e.g. vaccination) were performed (yes/no).

Section 3 contained questions on sociodemographic information such as age, gender, marital status, education, occupation and health-insurance status of the participants.

### Statistical analysis

Descriptive statistics such as frequencies, mean values and percentages were used for evaluating sample characteristics and analysing use-related variables such as HM indications or aims. To facilitate group comparisons, some categories of variables were merged, e.g. the sub-categories of former smokers and non-smokers were merged into one category of non-smokers, and current and occasional smokers into one category of smokers.

For testing group differences between the HM user in the previous 12 months (1), the HM user who did not take HM in the previous 12 months (2), and the non-user (3), Chi Square tests were performed and tested on the *p* < 0.05 significance level. Furthermore, binary hierarchical logistic regression analyses were used for identifying, in model 1, the relevant predictors for being a 12-month user (group 1) compared to a non-user (group 3) and, in model 2, for being a 12-month user (group 1) compared to being a less recent user (group 2). We decided that two separate regression models comparing two groups to each other, in a binary analysis, is more appropriate than an ordinal one because only for two out of the three groups could we enter “reasons for HM usage” as variables in the analysis. Consequently, due to the different number and sets of variables it was not possible to compare all three groups in one ordinal model.We applied a hierarchical model for analysing the incremental amount of variance that is explained when different sets of predictors (see below) are included successively in the model [25–27]. For both regression models, we used as the dependent variable “HM use in the last 12 months” (yes = 1/no = 0), excluding in model 1 the group of users who did not take HM in the previous 12 months (group 2) and in model 2 the group of non-users (group 3).

In both models, we entered as independent variables all sociodemographic variables (see Table 1) in a first block, and all health related variables (see Table 2) in a second block, which were found to be significant predictors in previous research on HM [15, 28–30]. We excluded the variable “marital status” to avoid collinearity, due to its high correlation with the variable “size of household”. Additionally, in model 2 we entered in a third block variables of user ratings measuring reasons for HM usage. Because addressing the reasons for HM usage was of course only relevant for the groups of users (1,2), these variables were only included in regression model 2. The results shown in Table 6 were those of the final hierarchical model, in which nominal scale variables were entered as k – 1 dummy variables. Note that the respective reference group is described in the table captions.

**Table 1:** Frequency table on sociodemographic characteristics of study participants and results of χ^2^ test

	Total sample, *n* = 2906 (%)	HM user in previous year (1), *n* = 2192 (%)	HM user not in previous year (2) *n* = 328 (%)	HM non-user (3), *n* = 386 (%)	χ^2^ (df), *p* value
Total N	100.0	75.4	11.3	13.3	
Gender					
Male	49.4	44.5	59.8	68.4	χ^2^(2) = 90.62, *p* = .000
Female	50.6	55.5	40.2	31.6	
Years of Education					
< 12 Years	50.1	47.4	54.9	60.9	χ^2^(2) = 27.12, *p* = .000
≥ 12 Years	49.9	52.6	45.1	39.1	
Occupation					
Employed	64.7	66.0	60.1	61.4	χ^2^(2) = 6.59, *p* = .037
Unemployed	35.3	34.0	39.9	38.6	
Health insurance					
Public	78.6	77.4	80.2	83.9	χ^2^(2) = 9.65, *p* = .047
Private	21.4	22.6	19.8	16.1	
Age group (years)					
18–29	16.2	17.6	13.7	10.6	χ^2^(8) = 17.39, *p* = .026
30–39	13.0	13.0	10.7	14.5	
40–49	17.2	17.0	18.0	17.9	
50–59	19.4	19.1	18.9	21.5	
≥ 60	34.2	33.3	38.7	35.5	
Size of household size					
1	25.2	23.0	30.8	32.9	χ^2^(6) = 31.37, *p* = .000
2	42.8	42.8	42.1	43.3	
3	15.7	16.7	13.7	11.7	
≥ 4	16.3	17.5	13.4	12.2	
Marital Status					
Married/cohabitating	51.8	53.0	47.3	49.0	χ^2^(6) = 7.55, *p* = .273
Divorced/separated	14.3	13.7	17.7	14.5	
Single	30.1	29.7	30.5	32.1	
Widowed	3.8	3.6	4.6	4.4

**Table 2:** Frequency table on health-related characteristics of study participants and results of χ^2^ test

	Total sample, *n* = 2906 (%)	HM user in previous year (1), *n* = 2192 (%)	HM user not in previous year (2) *n* = 328 (%)	HM non-users (3), *n* = 386 (%)	χ^2^ (df), *p* value
Self-perceived health status					
Very good, good	62.8	63.0	63.4	61.4	χ^2^(2) = .41, *p* = .813
Fair, poor, very poor	37.2	37.0	36.6	38.6	
Chronic disease					
Yes	53.6	56.6	48.2	41.5	χ^2^(2) = 35.57, *p* = .000
No	46.4	43.4	51.8	58.5	
Smoking Status					
Current	30.1	28.8	33.2	35.0	χ^2^(2) = 7.55, *p* = .023
Former, Never	69.9	71.2	66.8	65.0	
Regular Alcohol Consumption					
Yes	64.0	64.5	63.4	61.7	χ^2^(2) = 1.21, *p* = .545
No	36.0	35.5	36.6	38.3	
Physical Activities (d./week)					
0–2	57.0	54.6	62.2	65.8	χ^2^(2) = 20.93, *p* = .000
3–7	43.0	45.4	38.8	34.2	
Flu vaccination					
Yes	22.2	22.6	22.6	19.7	χ^2^(2) = 1.62, *p* = .445
No	77.8	77.4	77.4	80.3	
Annual dentist check-ups					
Yes	74.8	78.0	67.1	63.5	χ^2^(2) = 48.33, *p* = .000
No	25.2	22.0	32.9	36.5	
Preventive medical check-ups					
Yes	53.7	56.6	47.9	42.0	χ^2^(2) = 33.35, *p* = .000
No	46.3	43.4	52.1	58.0	
Current tetanus vaccination					
Yes	74.6	76.1	72.9	67.1	χ^2^(2) = 14.72, *p* = .001
No	25.4	23.9	27.1	32.9	

For all calculations, we used the statistics software package SPSS for Windows, release 23.

## Results

In this section, we first report the status quo of the HM usage in the general German population in regard to sociodemographic and health-related characteristics and usage pattern. In the last part of the results section, we report results on influencing factors for HM usage.

### Prevalence, sociodemographic and health-related characteristics

Table 1 shows the distribution of sociodemographic variables of the total sample as well as the comparison between the three groups. The sample included 2906 participants, of which 2192 people used HM in the previous 12 months (75.4% 12-month prevalence). 328 people did not use HM in the previous 12 months, but did so at earlier times (86.7% lifetime prevalence) and 386 participants had never used HM. Interestingly, 92% of the participants that had used HM in the previous year did so in self-medication, of which only 38% informed their physician. Results from Chi Square tests showed that HM users were mostly female, educated longer, employed and insured privately. Furthermore, statistically significant differences (*p* < 0.05) were found for the variables age and size of household. The variable marital status did not show differences between the groups.

The results for health-related variables are shown in Table 2. Results from Chi Square tests showed that recent HM users were more often suffering from a chronic disease, non-smokers, and performed physical activity more frequently, compared to the other groups. Statistically significant differences between the groups were also found in preventive health behaviour: recent HM users did more often annual dentist check-ups, preventive medical check-ups, and were more likely to be currently vaccinated against tetanus. The variables of flu vaccination, alcohol consumption and self-perceived health-status did not show differences between the groups.

### Usage pattern

For this part of the results section, we focus on the data for the participants who used HM in the previous year (group 1), in order to enable comparison of our findings to previous studies [12, 29]. Regarding the aims of HM usage, 93.1% of the participants used HM to treat current or chronic illnesses, 70.7% to promote health, and 60.9% to prevent illnesses. The most frequently used herbs were peppermint (58.4%), camomile (58.3%), sage (55.7%), ginger (51.9%), eucalyptus (46.3%), onion (33.2%), valerian (33.1%), stinging nettle (30.5%), St. John’s wort (30.3%), *Aloe vera* (28.4%), and arnica (28.4%). The most frequent conditions for which HM were used were common cold/flu infection (65.8%), respiratory problems (61.8%), and gastrointestinal diseases (47.8%). The perceived helpfulness of HM was greatest for insect bites, gastrointestinal and respiratory problems. Especially for tinnitus and depression, HM was not perceived as being very helpful. Frequencies of all indications for which HM was used and the reported helpfulness are shown in Table 3.

**Table 3:** Frequency table on indications for using HM and perceived helpfulness

INDICATIONS	N	%	PERCEIVED HELPFULNESS (%)		
			Not at all	Somewhat	A great deal
Common cold/flu infection	1441	65.8	2.3	56.8	40.9
Respiratory problems	1355	61.8	2.4	49.4	48.2
Gastrointestinal diseases	1048	47.8	2.0	44.8	53.2
Sleeping disturbances	655	29.9	15.7	53.4	30.9
Anxiety/restlessness	500	22.8	13.6	54.4	32.0
Insect bites/itching	437	19.9	3.9	33.9	62.2
Headaches/migraines	386	17.6	10.9	54.4	34.7
Musculoskeletal problems	353	16.1	12.8	60.6	26.6
Back/neck pain	348	15.9	12.4	58.9	28.7
Bruises/sprains	342	15.6	5.8	46.2	48.0
Chronic pain	334	15.2	10.2	62.0	27.8
Gynaecological/urological problems	311	14.2	7.1	48.2	44.7
Dermatosis	293	13.4	9.9	48.5	41.6
Allergies/hay fever	218	9.9	17.0	48.6	34.4
Depression	185	8.4	24.9	51.9	23.2
Blood pressure problems	169	7.0	14.2	59.2	26.6
Cardiovascular diseases	144	6.6	8.3	56.3	35.4
High cholesterol	89	4.1	18.0	57.3	24.7
Mental function	86	3.9	18.6	51.2	30.2
Tinnitus	68	3.1	52.9	33.8	13.3
Asthma	63	2.9	23.8	49.2	27.0
Diabetes	54	2.5	22.2	61.1	16.7
Cancer	33	1.5	24.2	45.5	30.3

*N* = 2192 (group of HM users in the last 12 months, see Table 1); multiple answers allowed

To the question “Where do you inform yourself about the effectiveness and possible areas of application of HM”, researching on the internet was the most frequent answer, followed by consultation of pharmacists, parents and family members, and physicians (see Table 4). Despite the fact that the internet was mentioned as the most popular source of information, only 25.4% of the participants also trusted this source. Much more trust is advocated to pharmacists (68.1%), physicians (58.1%), and as third frequent family members (28.5%). All results related to the sources of information are shown in Table 4.

**Table 4:** Frequency table on sources of information and trust in the source

SOURCE OF INFORMATION			TRUST IN SOURCE	
	N	%	N	%
Internet	1495	68.2	556	25.4
Pharmacist	1187	54.2	1492	68.1
Family members	1000	45.6	624	28.5
Physician	849	38.7	1274	58.1
Friends	760	34.7	318	14.5
Package insert	755	34.4	577	26.3
Books	668	30.5	528	24.1
Product package	655	29.9	353	16.1
Journals/magazines	592	27.0	254	11.6

*N* = 2192 (group of HM users in the last 12 months, see Table 1); multiple answers allowed

In Table 5 we show results regarding participants’ self-perceived knowledge about different topics concerning HM usage and their agreement whether or not they wish to be better informed. 35.3% of participants rated their knowledge about potential side effects as poor or very poor, compared to only 14.8% who thought to have good or very good knowledge about this topic. The situation is even more pronounced for potential interaction effects: 45.2% of the participants in group 1, i.e., almost half of the regular HM users, rated their knowledge on potential interaction effects as poor or very poor, and only 14.8% as good or very good. Moreover, it was found that in all topics essentially every other participant indicated that he/she wished he/she were better informed, and only 9.7% of the participants responded that they were informed well enough.

**Table 5:** Frequency table on self-perceived knowledge about HM

KNOWLEDGE	very poor, poor	moderate	good, very good	“Yes, I wish I were better informed”
	N (%)	N (%)	N (%)	%
Visual identification and differentiation of raw medicinal plants	986 (44.9)	842 (38.4)	364 (16.6)	41.0
Medicinal effect and areas of application of raw medicinal herbs	704 (32.1)	997 (45.5)	491 (22.3)	51.9
Medicinal effects and areas of application of processed HM products	469 (21.3)	1058 (48.3)	665 (30.3)	51.0
Potential unwanted side effects of raw or processed HM products	774 (35.3)	945 (43.1)	473 (21.6)	53.0
Potential unwanted interaction effects with other HM products	992 (45.2)	876 (40.0)	324 (14.8)	56.2
Safe dosage and safe use	361 (16.5)	908 (41.4)	923 (42.1)	43.4

Total *N* = 2192 (group of regular HM users, see Table 1); Question: “I wish I were better informed” allows for multiple answers

### Factors influencing HM usage

Within this section, we analyse which factors can influence HM usage, i.e., we report the results from binary hierarchic logistic regression analysis, applying two different models as explained in the methods section.

Regarding the overall model evaluation, both logistic models showed statistical significance of *p* = 0.000 in all likelihood-ratio tests, yielding the conclusion of model effectiveness compared to the respective null models. The Hosmer-Lemeshow-test for assessing the goodness of the model fit again showed reasonable results, as demonstrated by non-significance for both models in all hierarchical blocks (model 1: first block: χ^2^(8) = 6.291, *p* = .615; second block: χ^2^(8) = 5.397, *p* = .714); model 2: first block: χ^2^(8) = 8.785, *p* = .361; second block: χ^2^(8) = 12.282, *p* = .139; third block: χ^2^(8) = 9.516, *p* = .301). The overall correct classification rate was 85.1% for model 1 and 87% for model 2. The model evaluation, the goodness-of-fit statistics and the validation of the predicted probabilities demonstrated that both models fitted the data for distinguishing between the groups [25, 27, 31].

In regard to model 1, as shown in Table 6 it was found that HM usage in the last 12 months (group 1) compared to HM non-usage (group 3) was significantly and positively associated with the variables being female (as the strongest predictor), living in bigger households, being employed, being educated for 12 or more years, and having private health insurance. The sociodemographic variables explained 9.8% of variance of the dependent variable. Entering the health-related variables in a second block could improve the total amount of explained variance up to 15.3%, whereby having chronic diseases, engaging in physical activities more than 2 times per week, and going to annual dentist check-ups were found to be factors significantly and positively associated with HM usage. In model 2, the following sociodemographic variables were found to be significantly and positively associated with being a HM user in the last 12 months (group 1) compared to being a less recent HM user (group 2): being female, being younger, and living in bigger households. The sociodemographic variables explained a rather small part of the variance of only 3.8%. Entering the health-related variables could improve the prediction, explaining 6.2% of the variance. Having a chronic disease and going to annual dentist check-ups were found to be factors that were associated with a more recent HM use. Interestingly, including the ratings on different statements concerning reasons for HM usage within a third block of variables could improve the Nagelkerke R^2^ from .062 to .172 in model 2, which means the model could explain 17.2% of variance instead of 6.2%. To be specific, the more someone agreed to the following reasons, the more likely the person belonged to group 1, HM users in the last 12 months: “I take HM because chemically synthesised medicinal products have too strong side effects”, “I take HM because I had positive experiences with HM products”, “I take HM because they have had a positive impact on my health” and “I take HM because within my family we have always used HM”.

**Table 6:** Results of the two binary hierarchical regression analyses to identify influencing factors of herbal medicine use

	Model 1: HM user in previous year (1; *n* = 2192) vs. HM non-user (0; *n* = 386)				Model 2: HM user in previous year (1; *n* = 2192) vs. HM user not in prev. Year (0; *n* = 328)			
Independent Variable	B	Adj. OR	95% CI	R^2^	B	Adj. OR	95% CI	R^2^
Gender (Female)	1.060**	2.887	2.241–3.719		.537**	1.711	1.307–2.239	
Age 18–29	.364	1.439	.933–2.219		.537*	1.710	1.085–2.696	
30–39	−.157	.855	.562–1.299		.542*	1.720	1.053–2.810	
40–49	−.093	.911	.623–1.334		.094	1.098	.721–1.673	
50–59	−.285	.752	.532–1.064		.020	1.020	.691–1.506	
Size of household 2	.291*	1.337	1.014–1.763		.435**	1.544	1.136–2.099	
3	.752**	2.121	1.439–3.125		.431*	1.539	1.023–2.317	
≥4	.669**	1.952	1.326–2.872		.484*	1.623	1.070–2.461	
Occupation (Employed)	.278	1.320	.993–1.754		.148	1.160	.850–1.582	
Education (≥ 12 years)	.461**	1.585	1.240–2.027		.235	1.265	.967–1.653	
Health insurance (Private)	.463**	1.589	1.164–2.171	.098	.179	1.195	.868–1.647	.038
Self-perceived health status (fair, poor, very poor)	−.099	.906	.683–1.201		.238	1.269	.936–1.719	
Chronic disease (Yes)	.882**	2.416	1.836–3.178		.450**	1.568	1.176–2.090	
Smoking status (Former, Never)	.088	1.092	.851–1.402		.086	1.089	.827–1.435	
Regular alcohol consumption (No)	−.229	.795	.623–1.015		−.151	.860	.658–1.124	
Physical activities (≥3 d./week)	.431**	1.538	1.208–1.960		.192	1.212	.934–1.572	
Flu vaccination (Yes)	.083	1.087	.802–1.472		.124	1.132	.823–1.557	
Annual dentist check-ups (Yes)	.356**	1.427	1.096–1859		.350*	1.419	1.056–1.906	
Preventive medical check-ups (Yes)	.161	1.175	.905–1.525		.087	1.091	.824–1.444	
Current tetanus vaccination (Yes)	.175	1.192	.913–1.555	.153	−.038	.963	.712–1.301	.062
“I take HM because chemically synthesised medicinal products have too many side effects.”					−.131	.877	.726–1.060	
“I take HM because chemically synthesised medicinal products have too strong side effects.”					2.09*	1.232	1.024–1.484	
“I take HM because chemically synthesised medicinal products did not show treatment success.”					−.070	.932	.794–1.095	
“I take HM because I was dissatisfied with the conventional medical practitioner.”					.020	1.020	.876–1.188	
“I take HM because in the past, I had positive experiences with herbal medicinal products.”					.334**	1.397	1.155–1.689	
“I take HM because they have had a positive impact on my health.”					.417**	1.517	1.248–1.844	
“I take HM because they are healthier than chemically synthesised medicinal products.”					−.019	.982	.821–1.174	
“I take HM because they are more natural than chemically synthesised medicinal products.”					−.004	.996	.822–1.206	
“I take HM because they have a higher tolerability than chemically synthesised medicinal products.”					.127	1.136	.933–1.383	
“I take HM because they have less side effects than chemically synthesised medicinal products.”					−.078	.925	.752–1.139	
“I take HM because I trust HM more than chemically synthesised medicinal products.”					−.069	.933	.790–1.103	
“I take HM because within my family we have always used HM.”					.269**	1.308	1.121–1.527	
“I take HM because I am very familiar with herbal medicinal products since my childhood.”					−.096	.909	.788–1.047	.172

Note. Reference categories: gender: male; age: 60+, size of household: 1; occupation: non-employed; education: < 12 years; health insurance: public; self-perceived health status: very good, good; chronic disease: no; smoking status: yes; regular alcohol consumption: yes; physical activities (0–2 d./week); flu vaccination: no; annual dentist check-ups: no; preventive medical check-ups: no; current tetanus vaccination: no. B = regression coefficient b; Adj. OR = adjusted odds ratio. CI = confidence intervals for odds ratio. * *p* ≤ 0.05; **p ≤ 0 .01; R^2^ = Nagelkerkes Pseudo R^2^. Bold data are significant

## Discussion

In our study, we undertook a nationwide online survey on the general population in Germany to examine the role of HM in the German health-care system and to address important questions about the prevalence of HM usage, the users´ characteristics, usage pattern, and factors influencing the use.

Our first important finding concerns HM prevalence rates for the general population in Germany, which were found to be impressively high (75.4% 12-month prevalence, 86.7% lifetime prevalence) compared to those reported for other countries (e.g. 39.2% of the general population in Turkey [15], 63.5% in Kuwait [11], 36.8% in Australia [17] or 33.9% in Malaysia [14]). This finding is in line with the general trend of increasing prevalence rates of HM use in Germany, which can be observed comparing the 12-month prevalence rate of 26.6%, found by Härtel & Volger in 2004 [32], and 70% reported in 2010 [16]. All of these results underline the importance of HM in the German health-care system. Why HM prevalence rates in Germany are higher than in other countries, and why they are increasing is a question that may not have a simple answer. But one can assume that these observations are related to the cultural and social embedment, individual values and experiences guiding specific reasons for a high prevalence of HM usage. It could also be a consequence of specifics of the German health-care system (e.g. health insurance and social security policies) as well as advanced marketing activities. Future research is required to understand the frequent user decisions for HM as treatment method in the society.

Regarding the sociodemographic- and health-related variables, the German HM user was found to be more likely female, highly educated, living in bigger households, and more likely to be privately insured and employed. These results are mostly in line with previous, well-established findings on HM [12, 15, 29, 30, 33, 34], especially concerning the variables of gender and education. Analysing the health-related aspects, HM users more often suffered from a chronic disease than non-regular or non-users, and showed a lifestyle that was clearly more health-oriented: HM users engaged more in physical activity, were more often non-smokers, and performed preventive health behaviour (e.g. preventive medical check-ups). Again, these results are mostly in line with previous research, as similar group differences were found, for example, also in the context of physical activities [12], smoking behaviour [15], and suffering from a chronic disease [30]. Moreover, in our sample we could not find group differences regarding the self-perceived health status, which is in contrast to the findings of previous research [12, 29, 34].

In our study, in total 93% of regular HM users were found to take HM in self-medication, which again can be interpreted as another form of proactive health-oriented behaviour. Hence, for regular HM users, influencing and managing their own health seems to be an important issue. The high percentage of self-medication related to HM is a phenomenon that has been frequently discussed in literature, together with the fact that users do not inform their physician about their “self-prescription” [12, 15, 17, 31, 34–37]. Also in our study, we found that only 38% of respondents using HM in self-medication informed their physician about it, which is quantitatively similar to previous studies in Italy [33], the US [12], and Turkey [15]. As discussed in the literature, patients may furthermore overestimate the positive aspects of HM and may not be aware of existing potential side and interaction effects [37–41]. For example, Nur [15] found a clear lack of risk awareness: only 18% of HM users knew about possible interaction effects with other drugs, and Meier & Lappas found a bias towards using natural vs. synthetic drugs regardless of safety and efficacy [42]. Taking all these outcomes together with the findings that the internet and not medicinal experts were noted as the most important information source in our study, and that HM users are not well-informed about the safety pattern of HM, there are clear signals of potential harm of HM usage in Germany, that health-care service providers and medicinal professionals should be aware of. Therefore, medicinal practitioners and pharmacists should actively ask their patients about their use of HM and inform them about HM safety profiles and interaction effects. These issues may be important especially for elderly HM users, who often take a number of prescribed and non-prescribed drugs [35, 36, 43, 44].

Regarding results of the binary hierarchical regression analyses, we first of all note that the value of the total R^2^, and therefore the amount of variance of the dependent variable explained after entering the sociodemographic and health-related variables, were found to be low. It was 15.3% for model 1 and only 6.2% for model 2, despite the fact that the overall model evaluations and the goodness of fit tests showed satisfactory results. Unfortunately, most previous studies reporting data from such models did not provide results on R^2^, and thus we could not find a detailed discussion on this apparent issue in the literature. But we have found one study that reported similarly low R^2^ values for predicting HM use for children and adolescents in Germany [28]. Nevertheless, it should be stressed that using model 1 we found significant values for the variables of gender, size of household, occupation, level of education, health insurance, chronic disease, physical activities, and annual dentist check-ups in predicting regular HM usage. This confirms to a large degree results of studies that were conducted in other countries [9, 15, 20]. The fact that having private insurance is a significant predictor in Germany is plausible as it covers financial costs associated with HM use that public insurances does not. Using model 2 we found significant predictive power for being a 12-month user rather than a less recent HM user for the variables gender, age, size of household, chronic disease, and annual dentist check-ups. The fact that user ratings on reasons for usage of HM could improve the R^2^ by more than 50% up to a value of .172 (17.2% explained variance) is a very interesting result. It shows that for the decision making process regarding the proximity in time of HM usage (last year: yes/no), the reasons or motives of people played a decisive role. This clearly points to important further factors influencing HM usage that go beyond mere “classical” user variables, and may include individual experiences, beliefs, values, cultural and social background, and personality traits of users, which indicates several highly interesting topics to be explored in further research in the future.

Finally, we address potential shortcomings of our study: as was discussed throughout this text, a clear terminology is critical when studying HM usage. In the questionnaire of this study, it was ensured that a clear definition of HM was used, such that there would not be a bias for the participants. Nevertheless, we cannot exclude the possibility that some participants have had medicinal products in mind that were not within our definition of HM when answering the questionnaire. Furthermore, our study sample is potentially biased because it was an online-sample. This study design could affect sociodemographic characteristics in our sample and may impact our results, e.g., our finding that researching the internet was the most frequent source of information of HM users. Participants have actively chosen to participate in the survey, therefore, even if we did not explicitly include “interest in HM” as a criterion for selection in our sample, there could still be a slight bias towards oversampling participants that show a general interest in HM. In principle, this could lead to an overestimation of the HM prevalence rates. Lastly, our questionnaire included a retrospective question whether or not people used HM in lifetime or in the last 12 months. This may of course result in inaccurate information and, as a consequence wrong classification in the three user groups.

## Conclusion

HM use was found to be common and widespread in the general German population with high prevalence rates, which demonstrates that is plays a central role in the health-care system in Germany. Future research is needed to explore in detail the reasons for this steady increase and the high user rates: Indeed, the user of HM in the last 12 months, the HM user who did not take HM in the last 12 months, and the HM non-user were found to differ in terms of certain sociodemographic and health-related variables. But as our data and analyses showed, the prediction of HM usage in the previous year was found to be improved by inclusion of other variables that addressed the reasons for HM usage. Self-medication, small knowledge of interaction and side-effects of HM, and a lack of communication about the usage were found to be part of the HM usage pattern. Taken together, and in view of the high prevalence rates of HM, this bears harm for patients of which health-service providers should be aware of.

## Supplementary information

**Additional file 1.** Questions asked in the online survey. Questions and items that were asked in the online survey and are relevant to this study.

## Data Availability

The dataset is available from the corresponding author upon reasonable request.

## Abbrevations

CAM: Complementary and alternative medicine
HM: Herbal medicine
