# Supplementary material for: The importance of herbal medicine use in the German health-care system: prevalence, usage pattern, and influencing factors
Source: BMC Health Serv Res. 2019 Dec 10;19:952. doi: 10.1186/s12913-019-4739-0 (PMC6905107; doi:10.1186/s12913-019-4739-0)
Supplement: Supplementary file 1 — Additional file 1. Questions asked in the online survey. Questions and items that were asked in the online survey and are relevant to this study. [file 12913_2019_4739_MOESM1_ESM.docx]

**Questions asked in the online survey**

| We are interested in the usage of “herbal medicine” for your own health or illness. We define herbal medicine as all plant-derived products including their natural form, as well as pills derived from extracts. You may know the following examples: “Sinupret”, St. John’s wort, herbal medicinal teas, etc.  Have you *ever* used natural herbs for your own health or illness (e.g. medicinal teas, herbal medicinal products,...)? | |
| --- | --- |
| - Yes | - No |

| During the past 12 months, did you use natural herbs for your own health or illness  (e.g. medicinal teas, herbal medicinal products, ...)? | |
| --- | --- |
| - Yes | - No |

| Have you also used herbal medicine in self-medication in the last 12 months without prescription or recommendation by a physician? | |
| --- | --- |
| - Yes | - No |

| Have you informed the use of herbal medicine in self-medication with your primary care doctor or health care provider? | |
| --- | --- |
| - Yes | - No |

| Do you use herbal medicine due to one of the following aims? | | |
| --- | --- | --- |
| To promote my health? | - Yes | - No |
| To prevent illnesses? | - Yes | - No |
| To treat illnesses? | - Yes | - No |

| For what health problems, symptoms, or conditions did you use herbal medicine in the last 12 months?  (items randomized, multiple answers allowed) | How much do you think did the herbal medicinal product help to treat the health problems, symptoms or conditions? Would you say ... | | |
| --- | --- | --- | --- |
|  | Not at all | Somewhat | A great deal |
| - Common cold/flu infection |  |  |  |
| - Respiratory problems |  |  |  |
| - Gastrointestinal diseases |  |  |  |
| - Sleeping disturbances |  |  |  |
| - Anxiety/restlessness |  |  |  |
| - Depression |  |  |  |
| - Mental function |  |  |  |
| - Musculoskeletal problems |  |  |  |
| - Bruises/sprains |  |  |  |
| - Back/neck pain |  |  |  |
| - Headaches/migraines |  |  |  |
| - Chronic pain |  |  |  |
| - High cholesterol |  |  |  |
| - Gynaecological/urological problems |  |  |  |
| - Dermatosis |  |  |  |
| - Cardiovascular diseases |  |  |  |
| - Tinnitus |  |  |  |
| - Blood pressure problems |  |  |  |
| - Allergies/hay fever |  |  |  |
| - Insect bites/ itching |  |  |  |
| - Diabetes |  |  |  |
| - Asthma |  |  |  |
| - Cancer |  |  |  |
| - Other |  |  |  |

| Which of the following herbs have you used in the last 12 months in their raw or processed form? (items randomized, multiple answers allowed) |
| --- |
| - Hops |
| - St. John’s wort |
| - Valerian |
| - Ginkgo |
| - Stinging nettle |
| - Sage |
| - Camomile |
| - Dandelion |
| - Arnica |
| - Onion |
| - Ribwort |
| - Evening primrose |
| - Echinacea |
| - Ginseng |
| - Comfrey |
| - Ginger |
| - Peppermint |
| - Passionflower |
| - Teatree |
| - Eucalyptus |
| - Calendula |
| - Horse chestnut |
| - Holy thistle |
| - Grapple plant |
| - Lavender |
| - Aloe vera |
| - Other |
| - I don´t know |

| Do you use herbal medicine due to one of the following reasons? Please rate your level of agreement with the following reasons. (items randomized) | | | | | |
| --- | --- | --- | --- | --- | --- |
| I take HM because … | Strongly disagree | Disagree | Neither agree nor disagree | Agree | Strongly agree |
| .. chemically synthesised medicinal products have too many side effects. |  |  |  |  |  |
| .. chemically synthesised medicinal products have too strong side effects. |  |  |  |  |  |
| .. chemically synthesised medicinal products did not show treatment success. |  |  |  |  |  |
| .. I was dissatisfied with the conventional medical practitioner. |  |  |  |  |  |
| .. in the past, I had positive experiences with herbal medicinal products. |  |  |  |  |  |
| .. they have had a positive impact on my health. |  |  |  |  |  |
| .. they are healthier than chemically synthesised medicinal products. |  |  |  |  |  |
| .. they are more natural than chemically synthesised medicinal products. |  |  |  |  |  |
| .. they have a higher tolerability than chemically synthesised medicinal products. |  |  |  |  |  |
| .. they have less side effects than chemically synthesised medicinal products. |  |  |  |  |  |
| .. I trust HM more than chemically synthesised medicinal products. |  |  |  |  |  |
| .. within my family we have always used HM. |  |  |  |  |  |
| .. I am very familiar with herbal medicinal products since my childhood. |  |  |  |  |  |

| Where do you inform yourself about the effectiveness and possible areas of application of HM? (multiple answers allowed) |
| --- |
| - Physicians |
| - Pharmacists |
| - Books |
| - Journals/magazines |
| - Friends |
| - Family members |
| - Internet |
| - Package insert |
| - Product package |

| Which of the following sources do you trust most concerning information on herbal medicine?  (multiple answers allowed) |
| --- |
| - Physician |
| - Pharmacist |
| - Books |
| - Journals/magazines |
| - Friends |
| - Familiy members |
| - Internet |
| - Package insert |
| - Product package |

| How do you rate your knowledge about herbal medicine in the following areas: | | | | | | Yes, I wish I were better informed! |
| --- | --- | --- | --- | --- | --- | --- |
|  | Very poor | poor | moderate | good | Very good |  |
| Visual identification and differentiation of raw medicinal plants. |  |  |  |  |  |  |
| Medicinal effect and areas of application of raw medicinal herbs. |  |  |  |  |  |  |
| Medicinal effects and areas of application of processed HM products. |  |  |  |  |  |  |
| Potential unwanted side effects of raw or processed HM products. |  |  |  |  |  |  |
| Potential unwanted interaction effects with other HM products. |  |  |  |  |  |  |
| Safe dosage and safe use. |  |  |  |  |  |  |

| How do you rate your health in general? Is it.. | | | | |
| --- | --- | --- | --- | --- |
| - Very good | - Good | - Fair | - Poor | - Very poor |

| Do you have any longstanding illness or health problem (for more than 6 months) or a chronic disease?  Note: a chronic disease is a longstanding illness, which requires constant monitoring and treatment, e.g. diabetes, arthrosis, migraine, asthma, hay fever, … | |
| --- | --- |
| - Yes | - No |

| Do you smoke? |
| --- |
| - Yes, currently more days per week or daily. |
| - Yes, occasionally. |
| - No, but I´m a former smoker. |
| - No, I’m not a smoker and smoked less than 100 cigarettes in my life. |

| How often did you drink alcohol in the last 12 months? |
| --- |
| - Daily |
| - On 5-6 days per week |
| - On 3-4 days per week |
| - On 1-2 days per week |
| - On 2-3 days per month |
| - Once per month |
| - Less than once per month |
| - Not in the last 12 months, because I don’t drink alcohol |
| - Never, or only a few sipps in my lifetime |

| On how many days in a typical week do you engage in physical activities (e.g. Nordic walking, ball games, cycling, running, rowing) for at least ten minutes without a break in your spare time? |
| --- |
| ____days/week |

| Please select whether or not the following statements apply: | |
| --- | --- |
| Within the last 12 months, I received a flu vaccination. | - Yes - No |
| Within the last 12 months, I had a preventive dentist check-up. | - Yes - No |
| I joined the last recommended preventive medicinal check-up (e.g. cancer screening, birthmark control …). | - Yes - No |
| I´m currently vaccinated against tetanus. | - Yes - No |

| I´m.. | |
| --- | --- |
| - male | - female |

| How old are you? |
| --- |
| - 18-29 years |
| - 30-39 years |
| - 40-49 years |
| - 50-59 years |
| - 60+ years |

| Marital status: |
| --- |
| - Married, living together with my spouse |
| - Married, living separated from my spouse |
| - Civil union, living together with my spouse |
| - Civil union, living separated from my spouse |
| - Single |
| - Divorced |
| - Widowed |

| What is your highest educational achievement? |
| --- |
| - Without graduation |
| - Secondary modern school or equivalent |
| - Graduation from polytechnical school in GDR |
| - School leaving graduation |
| - Bachelor |
| - Diploma |
| - Master |
| - Ph.D. |
| - Alternative degree:______________ |

| Are you currently employed? |
| --- |
| - Yes |
| - No |

| How many people live in your household constantly, including yourself? Please count all people that you are living together with, and please also include children. |
| --- |
| - One person |
| - Two people |
| - Three people |
| - Four people and more |

| Please indicate your health-insurance: |
| --- |
| - Private insurance |
| - Public insurance |
